# Predicting drug-metagenome interactions: Variation in the microbial β-glucuronidase level in the human gut metagenomes

**DOI:** 10.1371/journal.pone.0244876

**Published:** 2021-01-07

**Authors:** Moamen M. Elmassry, Sunghwan Kim, Ben Busby

**Affiliations:** 1 National Center for Biotechnology Information, National Library of Medicine, National Institutes of Health, Bethesda, MD, United States of America; 2 Department of Biological Sciences, Texas Tech University, Lubbock, TX, United States of America; University of Illinois, UNITED STATES

## Abstract

Characterizing the gut microbiota in terms of their capacity to interfere with drug metabolism is necessary to achieve drug efficacy and safety. Although examples of drug-microbiome interactions are well-documented, little has been reported about a computational pipeline for systematically identifying and characterizing bacterial enzymes that process particular classes of drugs. The goal of our study is to develop a computational approach that compiles drugs whose metabolism may be influenced by a particular class of microbial enzymes and that quantifies the variability in the collective level of those enzymes among individuals. The present paper describes this approach, with microbial β-glucuronidases as an example, which break down drug-glucuronide conjugates and reactivate the drugs or their metabolites. We identified 100 medications that may be metabolized by β-glucuronidases from the gut microbiome. These medications included morphine, estrogen, ibuprofen, midazolam, and their structural analogues. The analysis of metagenomic data available through the Sequence Read Archive (SRA) showed that the level of β-glucuronidase in the gut metagenomes was higher in males than in females, which provides a potential explanation for the sex-based differences in efficacy and toxicity for several drugs, reported in previous studies. Our analysis also showed that infant gut metagenomes at birth and 12 months of age have higher levels of β-glucuronidase than the metagenomes of their mothers and the implication of this observed variability was discussed in the context of breastfeeding as well as infant hyperbilirubinemia. Overall, despite important limitations discussed in this paper, our analysis provided useful insights on the role of the human gut metagenome in the variability in drug response among individuals. Importantly, this approach exploits drug and metagenome data available in public databases as well as open-source cheminformatics and bioinformatics tools to predict drug-metagenome interactions.

## Introduction

Over recent decades, a revolution in sequencing and characterization of human and environmental metagenomes [1,2] has made it possible to identify microbial enzymes that can metabolize our diet [3]. An emerging interdisciplinary area, called pharmacomicrobiomics [4,5], studies the effects of human-associated microbiomes on drugs (e.g., the effects of microbial enzymes on the bioactivity, bioavailability, and toxicity of drugs) [6]. Understanding the role of the microbiome in medication metabolism (pharmacokinetics) and its effect on the body’s response to these medications (pharmacodynamics) is necessary to optimize drug efficacy and safety. Although there is a systematic description of human enzymes that process different drugs, such information for bacterial enzymes does not exist yet [7]. Moreover, gut microbiome dysbiosis and dysfunction may have significant implications since certain disease states predispose patients to major drug interactions with the gut microbiota, as reported in a recent study in colorectal cancer patients [8].

**Fig 1:**
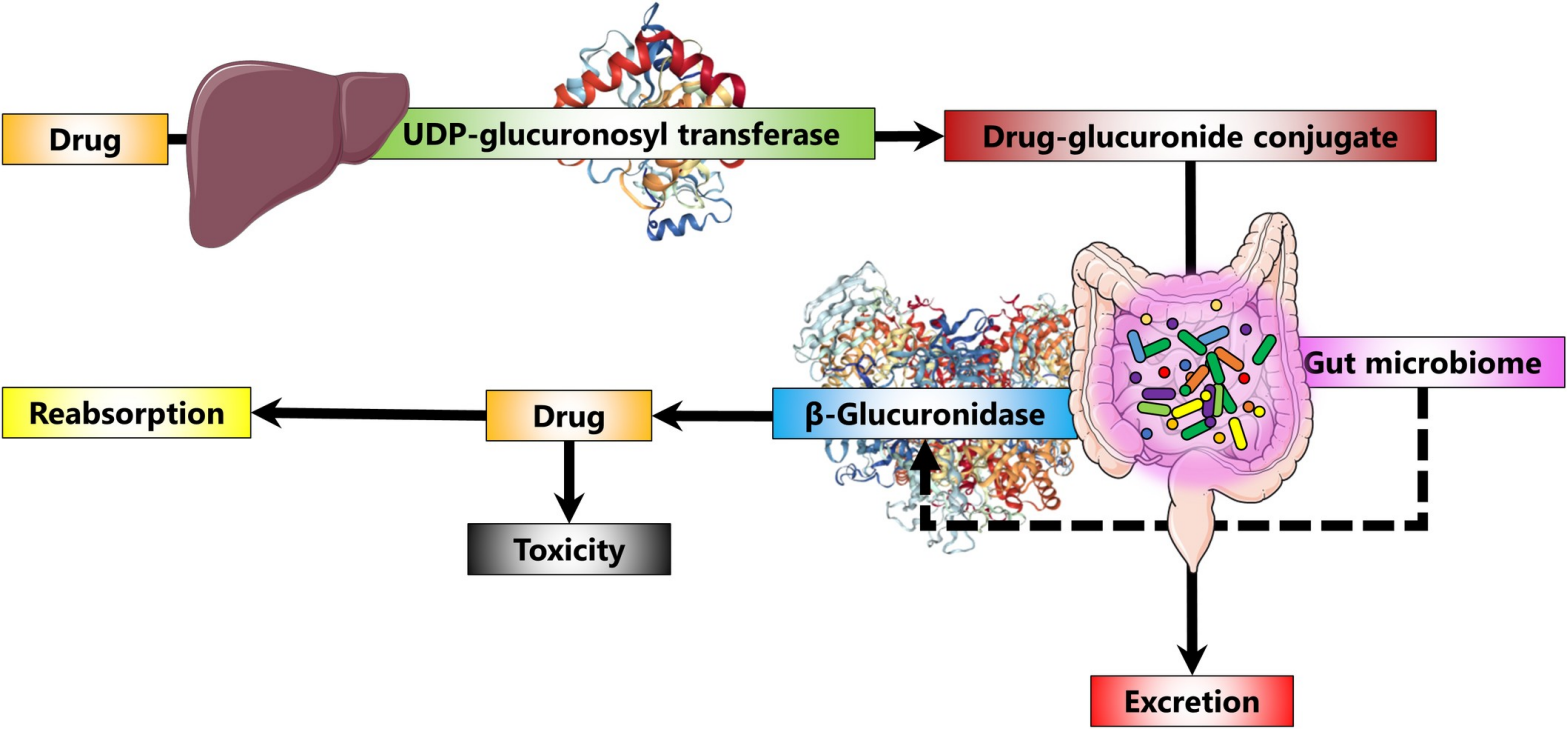
How gut microbiome affects the metabolism of glucuronidated drugs. Whether the drug is endogenous or exogenous, it is influenced by enterohepatic recirculation. In the liver, drugs (and their metabolites) are converted into drug-glucuronide conjugates through glucuronidation by uridine diphosphate (UDP)-glucuronosyl transferases. These conjugates are excreted via the intestinal tract. However, gut microbial β-glucuronidases can hydrolyze these conjugates into free drugs again, which can be reabsorbed into the systemic circulation.

Glucuronidation by uridine diphosphate (UDP)-glucuronosyl transferases is a major metabolic pathway that improves the elimination of many medications [9]. Through glucuronidation, many xenobiotics such as drugs (and their metabolites) form conjugates with glucuronides, which makes them more soluble and easier to be cleared from the body (Fig 1). On the other hand, β-glucuronidase can break down these drug-glucuronide conjugates, converting them back to the non-conjugated forms. For example, irinotecan, a chemotherapeutic agent, causes severe diarrhea in humans and animal models. This toxicity is due to the reactivation of its metabolite, SN-38, through the breakdown of the SN-38-glucuronide conjugate by bacterial β-glucuronidase [10,11]. This information is important for clinicians to predict drug response in individual patients and optimize drug dosage accordingly. The variability in the level of this enzyme in the gut among individuals has been investigated for a relatively small number of individuals (for example, 20 colorectal cancer patients [8] and 139 healthy volunteers [12]). Here, we used β-glucuronidase in the human gut microbiome as an example to present a novel computational approach to characterize and compare different human metagenomes for their capacity to harbor β-glucuronidases. We achieved this by exploiting public databases as well as open-source cheminformatics and bioinformatics tools. We presented 100 drugs for which their metabolism may be affected by microbial β-glucuronidase in the human gut. Evidence supporting this hypothesis already exists for many of them. We were able to generate hypotheses and predictions regarding metagenome-medication interactions that need to be experimentally tested to achieve drug efficacy and safety.

## Methods

### Collection of drugs potentially metabolized by β-glucuronidase

The DrugBank database (version 5.1.1) is a freely accessible online database, which contains comprehensive information on more than 11,000 FDA-approved and investigational drugs as well as their drug targets and metabolism [7,13]. DrugBank was used to collect drugs whose metabolism involves glucuronidation in the liver (S1 Data). This was accomplished by searching for drugs whose metabolism involves UDP-glucuronosyl transferase. It is noteworthy that β-glucuronidase affects the metabolism of these drugs in different degrees, because of several factors. First, the degree of glucuronidation differs between drugs. Second, some drugs are more readily transported to the gut through enterohepatic circulation [14]. Third, glucuronidated drugs can be terminally excreted in the urine, not only in the feces. Finally, different glucuronidated drugs can be impacted by β-glucuronidase variants at different rates due to enzyme-substrate specificity [12,15].

### Structural similarity analysis of chemical compounds

Structural similarity among the drug molecules identified from DrugBank was evaluated using the maximum common substructure (MCS) algorithm [16] and the Tanimoto coefficient (S2 Data), as defined in Eq (1). The Tanimoto coefficient between molecules A and B is defined as the following equation:

$$Tanimoto=\frac{c}{a+b-c}$$ (1)

where *a* and *b* are the total number of atoms in A and B, respectively, and *c* is the number of atoms in their MCS [16]. This was done using the *fmcsR* package [16], *ChemmineR* package [17], and R software version 3.5.2 [18]. The molecular structures necessary for the similarity computation were downloaded in structure data file (SDF) format through the PubChem Download Service [19,20], using the PubChem compound identifiers (CIDs) as input identifiers (obtained from DrugBank). The MCS algorithm was used to identify the largest substructure shared between compounds [16]. One mismatch of atoms and bonds were tolerated in the identified aromatic MCSs. Then, a similarity matrix of the Tanimoto coefficients—computed based on MCSs—between all medications was generated. Network analysis was performed based on the Tanimoto coefficients, and visualized using the following R packages: *reshape* [21], *tidyverse*, *tidygraph*, *ggraph*, and *igraph*. Example codes of the bioinformatics analysis scripts and packages used in R performed in the current study are available on GitHub [22].

### Preparation of human gut metagenome data sets

**Fig 2:**
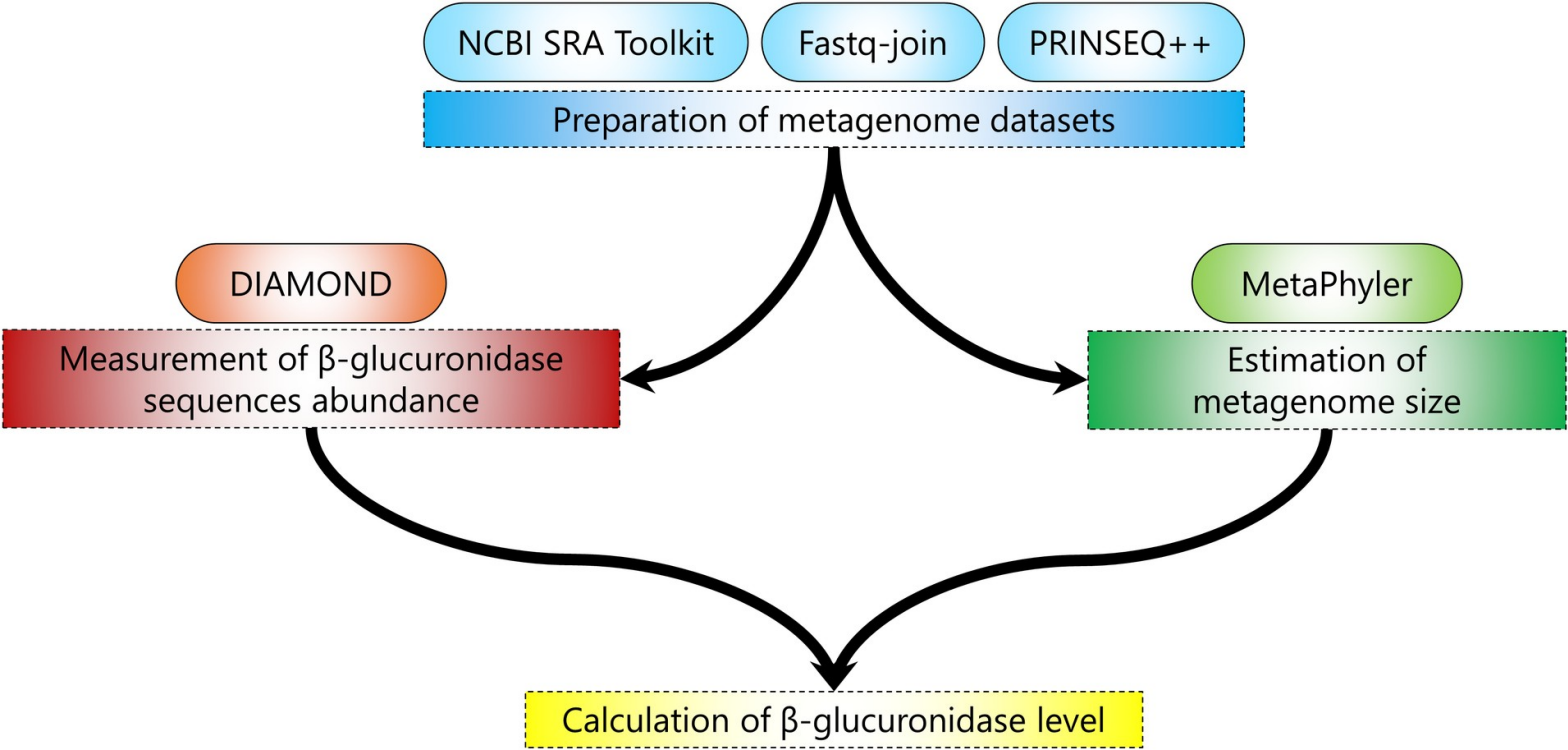
Schematic diagram of the main workflow used to estimate the β-glucuronidase level. The workflow shows the steps used to calculate the β-glucuronidase level in a metagenome. Rectangles with dashed borders show the main steps, while the rectangles with rounded corners and solid borders show the tools used in each step.

Fig 2 shows a schematic diagram of the main workflow for computing the variability in the microbial β-glucuronidase level in the human gut metagenome. The human gut metagenome data sets used in this study were downloaded from the Sequence Read Archive (SRA) [23,24], an international public repository for next-generation sequence data at the U.S. National Center for Biotechnology Information (NCBI). The lists of the SRA records used in this study are provided as S3 Data for male-vs.-female analysis and as S4 Data for infants-vs.-mothers analysis.

To compare the β-glucuronidase sequence abundance between males and females we used SRA records from the human microbiome project (HMP), PRJNA48479 [25]. The data set included samples of healthy adult gut metagenomes. The SRA records were collected using the SRA Run Selector, by filtering the HMP samples to only include SRAs with “G_DNA_Stool” as the analyte type and “PAIRED” as the LibraryLayout. The filtered data set contained 1793 metagenome samples (997 male samples and 796 female samples). The identifiers for these SRA samples are listed in S3 Data.

To compare the sequence abundance between infants and mothers, we used all SRA from the PRJEB6456 [26]. These SRA records comprise 400 gut metagenome samples: 300 samples taken from 100 infants at three different times (at birth, four months old, and one year old) and 100 samples from their mothers. The identifiers for these SRA samples are listed in S4 Data.

The paired-ends FASTQ files were retrieved for each SRA through the NCBI SRA Toolkit version 2.9.2 [27], using basic filtering options and the arguments listed in S5 Data. Then, forward and reverse reads were joined using Fastq-join version 1.3.1 [28,29]. The joined reads were quality filtered and converted from FASTQ to FASTA using PRINSEQ++ version 1.2 [30,31] with the arguments listed in S5 Data. Example codes of the software used in this section are available on GitHub [22].

### Estimation of gut metagenome size

In order to compare the different SRA samples for their β-glucuronidase sequence abundance, it is necessary to normalize our results to the metagenome size. To estimate the metagenome size we used MetaPhyler version 1.25 [32,33]. MetaPhyler is a taxonomic classifier for metagenomic shotgun reads that uses a set of universal, single-copy phylogenetic marker genes as a taxonomic reference [34]. We used the “blastn” option, which is recommended for short reads. We provide an example code for using MetaPhyler on GitHub [22].

### Calculation of β-glucuronidase level in gut metagenome samples

For alignment purposes, a protein BLAST database of representative β-glucuronidase sequences was created, S6 Data. These sequences were collected using the Conserved Domain Database (CDD) [35,36]. This was done using the retrieval function of representative sequences in the CDD for the sequences that harbor the domain that is present in known β-glucuronidases (PRK10150). PRK10150 was chosen because it is harbored by the proteins identified previously as β-glucuronidases [12].

DIAMOND (version 0.9.22.123) [37] was used to align short translated DNA sequences in each metagenome against a reference BLAST database of protein sequences. The number of sequences hit for each SRA was normalized based on SRA’s size estimated using MetaPhyler. The β-glucuronidase level was calculated as the following:

$$\beta ‐Glucuronidase\;level=\frac{BG}{T}\cdot 1000$$ (2)

where *BG* is the count of unique alignments to the β-glucuronidase database using DIAMOND and *T* is the metagenome size estimated using MetaPhyler (Fig 2).

Downstream analysis was performed using the *plyr* [38] package in R software version 3.5.2 [18]. *ggplot2* [39] and GraphPad Prism 7.0 were used to generate the density and box plots. For correlation analysis, the packages *reshape2*, *Hmisc*, and *stats* were used. Example codes used in this section are available on GitHub [22].

## Results

### The metabolism of a hundred medications is possibly altered by microbial β-glucuronidase

To find medications potentially affected by microbial β-glucuronidase, medications that can be metabolized to drug-glucuronide conjugates by UDP-glucuronosyl transferases were collected from the DrugBank database [7,13], a public database providing comprehensive information on Food and Drug Administration (FDA)-approved and investigational drugs. UDP-glucuronosyl transferases, central to the metabolism of xenobiotics, transfer a glucuronic acid to lipophilic compounds, thus making them more water-soluble to be readily excreted [9] (Fig 1). These xenobiotic-glucuronide conjugates are susceptible to breakdown by microbial β-glucuronidase, resulting in the release and reabsorption of the free xenobiotic in the gut (Fig 1).

**Fig 3:**
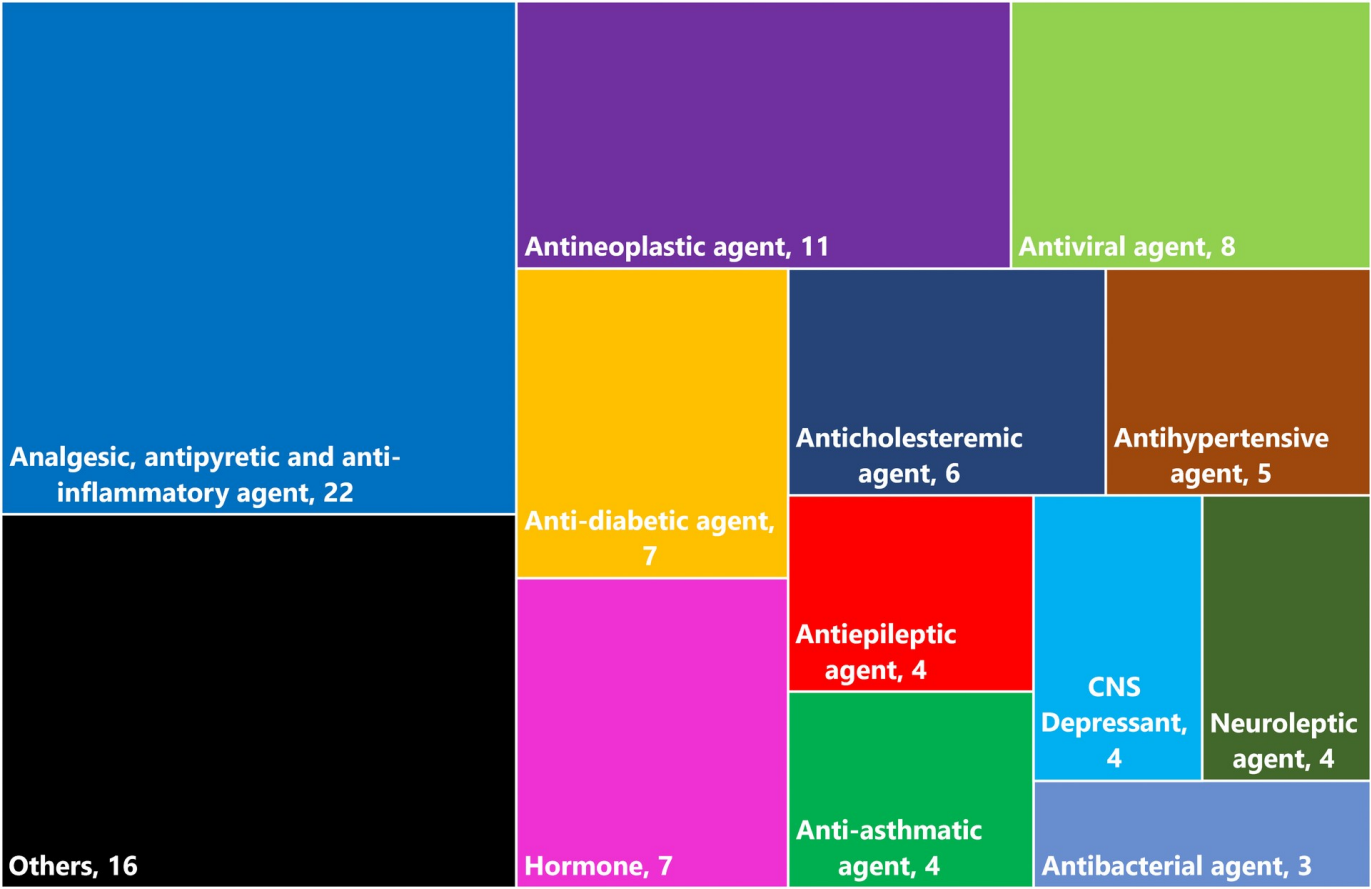
Treemap showing the therapeutic categories of the 100 medications whose metabolism involves glucuronidation and is possibly affected by β-glucuronidase.

We found 100 medications (S1 Data) known to be metabolized by glucuronidation and therefore potentially reactivated by microbial β-glucuronidase. These drugs are used for various treatments including pain management, chemotherapy, diabetes, hormonal therapy, hypercholesterolemia, acquired immunodeficiency syndrome (AIDS), central nervous system (CNS) disorders, hypertension, and asthma (Fig 3). The largest group of medications were 22 common pain management medications. This group included both over-the-counter (OTC) drugs (e.g., acetaminophen and ibuprofen) as well as prescription drugs (e.g., morphine).

### One-quarter of the medications potentially metabolized by β-glucuronidase are opioids, estrogens, NSAIDs, benzodiazepines, antihypertensives, and antidiabetics

**Fig 4:**
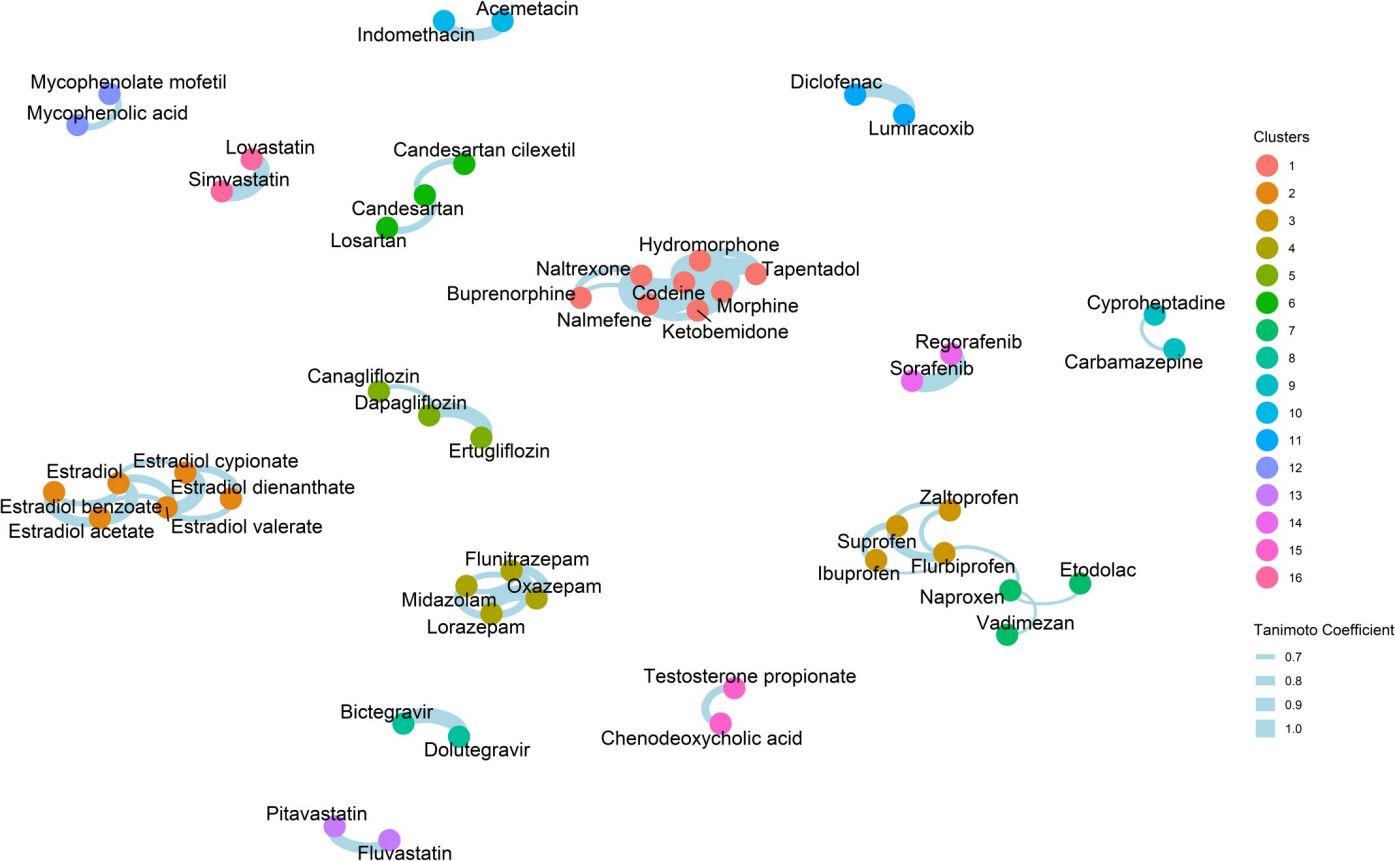
Structural similarity analysis of the 100 medications that can be influenced by the gut microbial β-glucuronidase. A network analysis of the 100 drugs was generated using their structural similarity, quantified using the maximum common substructure (MCS) algorithm and the Tanimoto Coefficients (Eq (1)). Nodes represent drugs and the edge between two nodes indicates a Tanimoto Coefficient of ≥ 0.65 between the drugs represented by the nodes Singletons are not shown. The complete similarity score matrix is provided in S2 Data.

**Fig 5:**
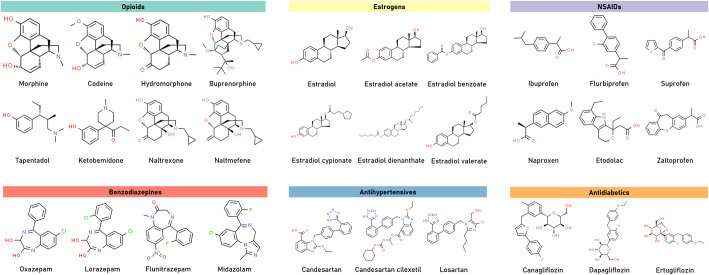
Chemical structures of the drugs contained in large clusters (with three drugs or more).

We were interested in whether any structural similarity exists between those 100 medications. To investigate this, we used the maximum common substructure (MCS) algorithm, which is a common approach used to identify the largest substructure (subgraph) shared between two compounds [16]. The MCS results were used to compute the Tanimoto coefficients between the drugs, which is commonly used to quantify molecular similarity (S2 Data) [16,40]. To identify common structural scaffolds, we generated a network of the 100 drugs (Fig 4), in which the nodes represent individual drugs and the edge between two nodes indicates a Tanimoto coefficient of ≥ 0.65 between the corresponding drugs. In the resulting network, the 100 drugs were grouped into 16 clusters (after removing singletons with only one compound). The structures of the drugs that belong to large clusters (containing three drugs or more) are shown in Fig 5. The largest cluster consisted of eight opioids including morphine, codeine, hydromorphone, naltrexone, nalmefene, tapentadol, ketobemidone, and buprenorphine (Figs 4 and 5). The second-largest cluster—consisted of two sub-clusters—had seven medications; six of which were non-steroidal anti-inflammatory drugs (NSAIDs) such as ibuprofen, flurbiprofen, suprofen, naproxen, zaltoprofen, and etodolac, and the antineoplastic agent, vadimezan. The third-largest cluster contained six compounds of estradiol and its different forms. The fourth-largest cluster was composed of the four benzodiazepine compounds: midazolam, lorazepam, oxazepam, and flunitrazepam. Among clusters that contained more than two compounds were the antihypertensives: candesartan, candesartan cilexetil, and losartan, and the antidiabetics: canagliflozin, dapagliflozin, and ertugliflozin.

### Estimation of the β-glucuronidase level in gut metagenomes

To examine the variability in the level of the gut microbial β-glucuronidase among individuals, human gut metagenome data sets were downloaded from the Sequence Read Archive (SRA) [23], as described in the Methods section. These data sets were analyzed using DIAMOND [37,41] and MetaPhyler [32,33]. DIAMOND is a well-established and memory-efficient tool for aligning protein or short translated DNA sequences against a reference database of protein sequences. We chose this approach as using translated DNA sequences is advantageous to using DNA sequences especially in aligning coding sequences mainly due to its sensitivity [42]. MetaPhyler was used [32,33] to estimate the metagenome size of each SRA record and normalize DIAMOND results. This normalization step was integral to our analysis to estimate the size of the microbial community and accurately adjust the β-glucuronidase level in each sample [34].

### Male and female gut metagenomes show significant variability in the β-glucuronidase level

**Fig 6:**
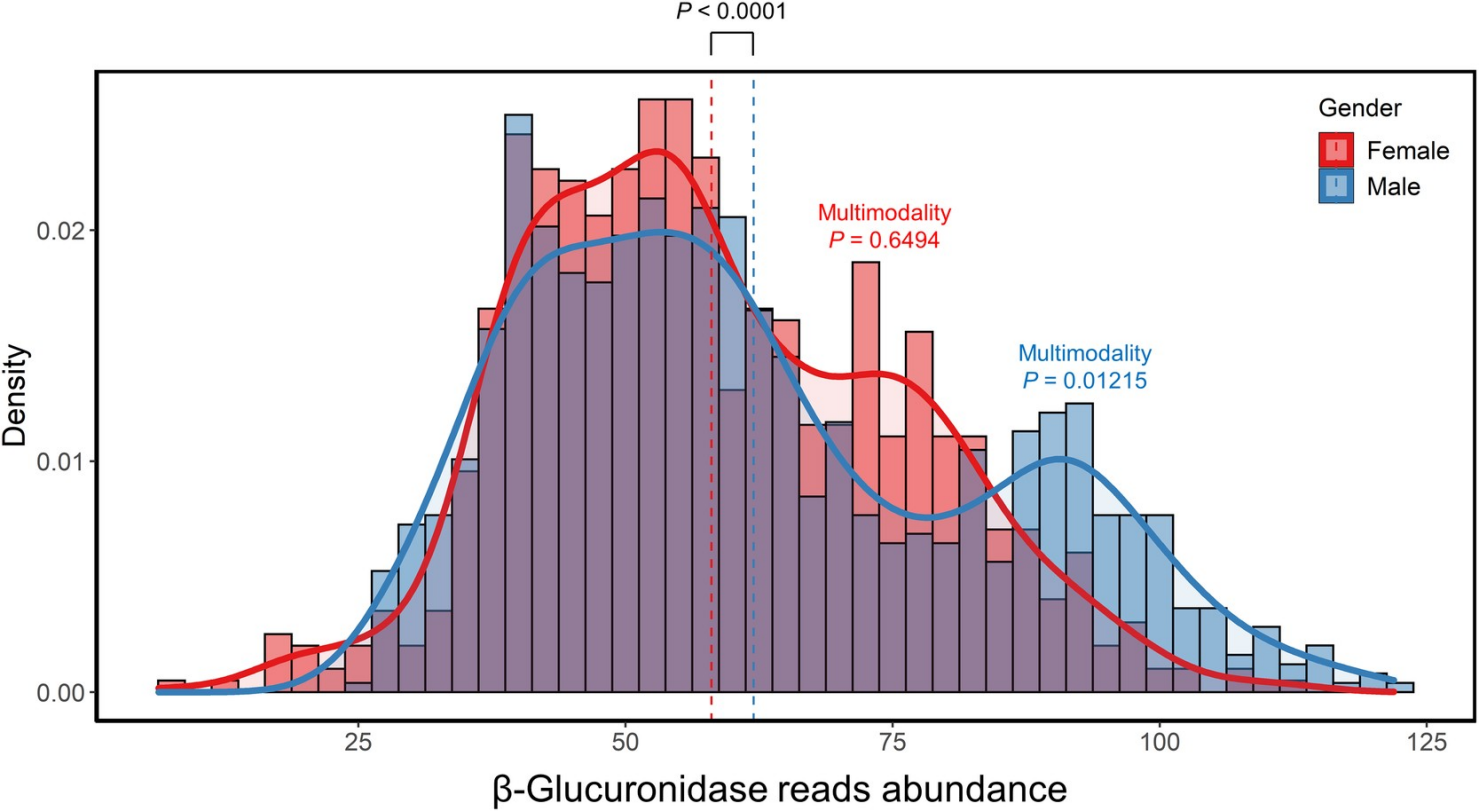
Density plot of the abundance of β-glucuronidase sequences in the 1793 gut metagenome samples from the human microbiome project (997 males and 796 males). The abundance of β-glucuronidase sequences was determined using DIAMOND.

As previously mentioned, estrogens were among the most commonly occurring structural classes in the 100 drugs that may be affected by microbial β-glucuronidase (Fig 4). Because of the role of estrogens as sex hormones in female biology, we were interested in the variation in the level of microbial β-glucuronidase between and within genders. Therefore, we analyzed 1793 gut metagenome samples (i.e., 997 males and 796 females) from the Human Microbiome Project (HMP) (BioProject: PRJNA48479, see the Methods section). We observed a slight, but statistically significant, increase in the mean β-glucuronidase level in male gut metagenomes than in female’s using Welch's t-test [43,44] (*P* < 0.0001; Fig 6). Furthermore, we observed a wide variability in the β-glucuronidase level among both males and females. Because the curves in Fig 6 showed multiple peaks that may arise from multiple subpopulations, we hypothesized that the β-glucuronidase level distribution within each gender group shows multimodality. This hypothesis was tested using the Hartigans' dip test [45] for unimodality/multimodality. The β-glucuronidase level distribution in male samples revealed multimodality (*P* = 0.01215). However, female samples did not show multimodality (*P* = 0.6494).

### Newborns and one-year-old infants gut metagenome harbor higher β-glucuronidase level than their mothers

**Fig 7:**
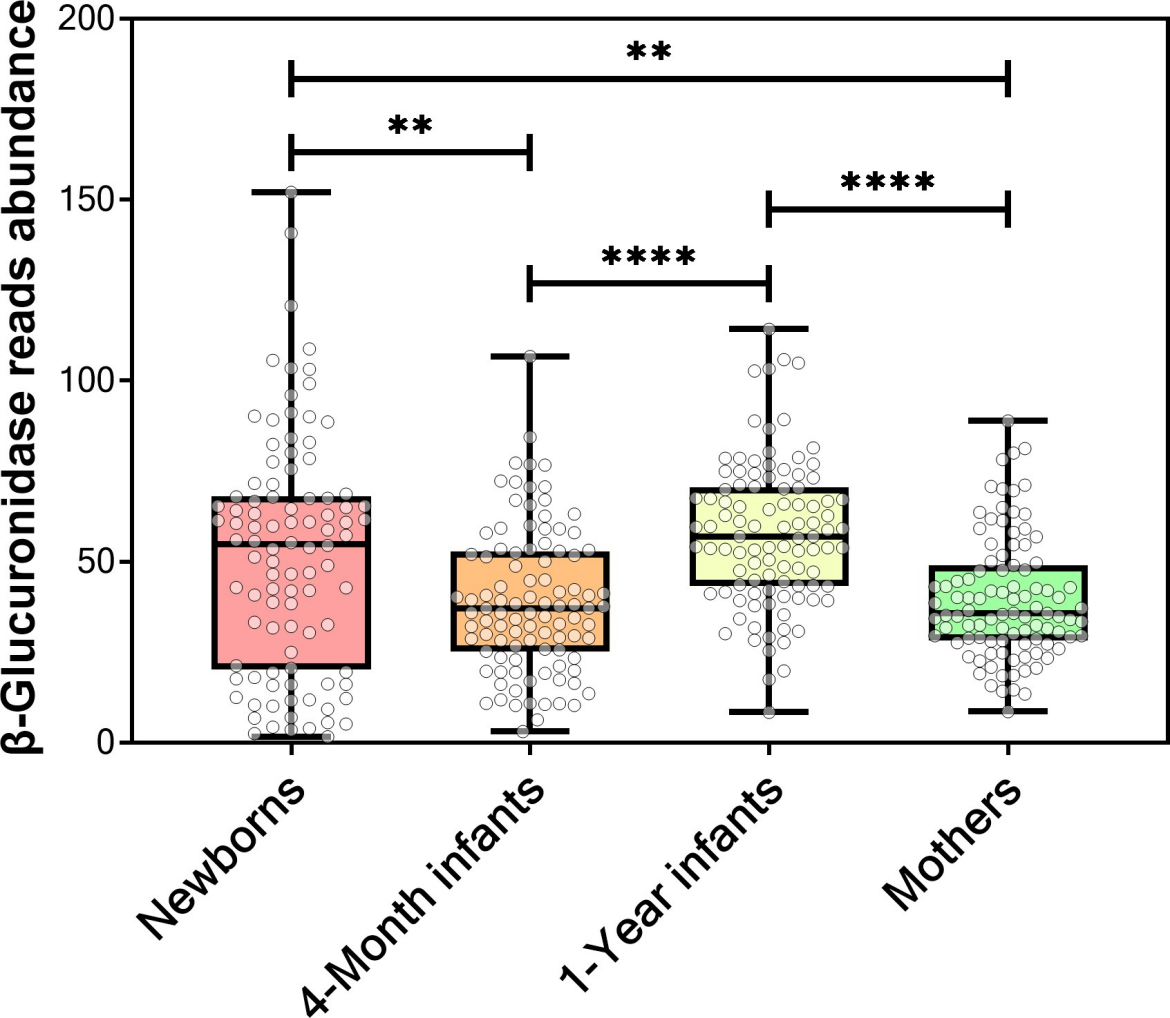
Box plot of the number of β-glucuronidase sequences in gut metagenomes of adults and infants. The number of β-glucuronidase sequences in mothers and infant gut metagenomes was calculated using DIAMOND (**P < 0.01 and ****P < 0.0001). The abundance of β-glucuronidase sequences was determined using DIAMOND.

β-Glucuronidase is involved in the breakdown/reactivation of bilirubin, which is a chemical generated during the normal breakdown of red blood cells [46–50]. Because of this, β-glucuronidase was suspected of its role in the development of hyperbilirubinemia in infants [46–50]. However, previous studies focused on the β-glucuronidase activity in breast milk and concluded that it is possibly a contributing factor but not the determining factor [46–50]. Therefore, we were interested in investigating the β-glucuronidase level in the infant metagenome. We analyzed the data set reported in a recent study by Bäckhed et al. (BioProject: PRJEB6456, see the Methods section) [26]. This data set consists of 400 gut metagenome samples: 300 samples taken from 100 infants at three different development stages (at birth, four months, and one year) and 100 samples from their mothers. We found that the gut microbial β-glucuronidase level was higher in the newborns and one-year-old infants compared to in their mothers, using the Kruskal–Wallis test followed by Dunn’s multiple comparisons test [51,52] (*P* < 0.01 and *P* < 0.0001, respectively) (Fig 7). Moreover, β-glucuronidase abundance was lower in four-month-old infants than newborns or one-year-old infants (*P* < 0.01 and *P* < 0.0001, respectively) (Fig 7). Therefore, we speculate that gut β-glucuronidase level change through infant development could be a factor contributing to bilirubin metabolism.

### *Bacteroides* strongly correlates with the β-glucuronidase level

**Fig 8:**
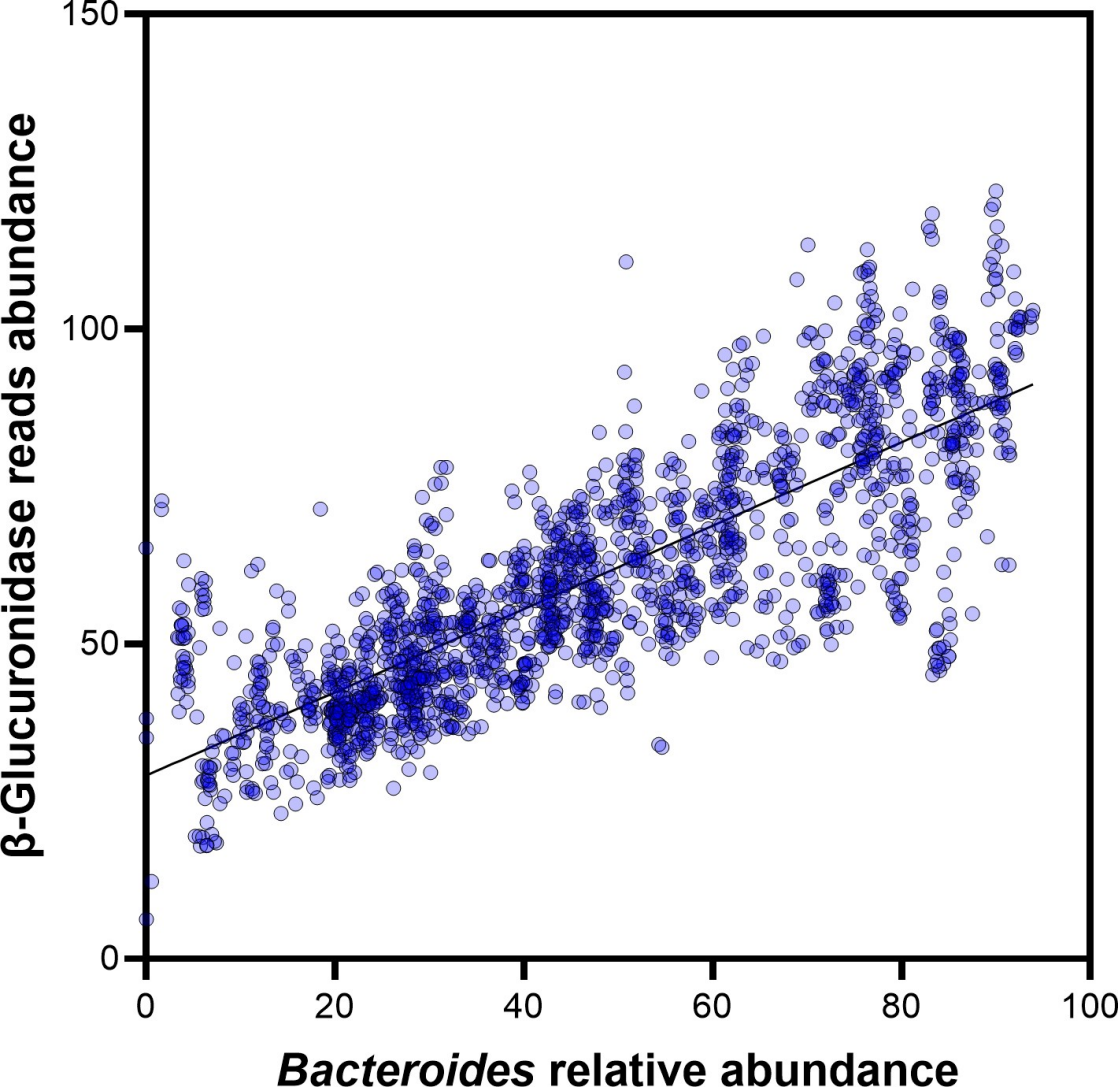
β-glucuronidase sequence abundance strongly correlates with *Bacteroides* relative abundance. Correlation between Bacteroides relative abundance and β-glucuronidase sequence abundance was observed in the gut metagenome data from the HMP. The nonparametric Spearman correlation coefficient (r) was 0.824 (*P* < 0.0001).

Several bacteria are known to encode numerous β-glucuronidases, thus we wanted to determine whether the observed β-glucuronidase abundance in the HMP gut metagenome data was correlated to a specific microbe. Therefore, we used MetaPhyler taxonomy results at the genus level to calculate the nonparametric Spearman correlation coefficient between the β-glucuronidase level and the relative abundance of each of the 200 genera identified in the HMP samples (S7 Data). Only the genus *Bacteroides* showed a strong correlation with the β-glucuronidase level (Fig 8) with *r* = 0.824 and *P* < 0.0001, after controlling for false discovery rate using Benjamini–Hochberg procedure [53].

## Discussion

Although human genomes play an important role in the response to a drug, it does not fully explain the variation in drug response among individuals [54]. The field of pharmacomicrobiomics provides new insights on the drug response variability beyond pharmacogenomics. In addition, pharmacomicrobiomics is receiving much attention from the biomedical science community because our microbiome can be easier to manipulate than our genomes [55]. To be able to manipulate our microbiome, it is imperative to characterize medications influenced by the metabolic capacity of the human microbiome, as well as uncovering microbial enzymes involved in drug metabolism.

The gut microbiota is a diverse microbial community with metabolic versatility that can affect the metabolism of hundreds of medications, as previously documented [56,57]. However, no systematic characterization of microbial enzymes that process particular classes of medications or a list of medications that are metabolized by such enzymes were presented. For example, the β-glucuronidase activity has been reported to cause severe toxicity for several medications (such as irinotecan [10,11,58], diclofenac [59], mycophenolate mofetil [60], and regorafenib [61]) and it has been demonstrated that this toxicity can be reduced by co-administration of β-glucuronidase inhibitors [59,61]. However, one may wonder if there are other drugs that could be affected by β-glucuronidase. One of the goals of this study is to suggest a potential approach to answer this question.

### Drugs affected by microbial β-glucuronidase

The present study identified 100 drugs known to form drug-glucuronide conjugates, which may be metabolized by microbial β-glucuronidase (S1 Data). These drugs were clustered into several groups according to their structural similarity (Fig 4). Among the resulting clusters, the largest ones contained opioids, estrogens, NSAIDs, benzodiazepines, antihypertensives, and antidiabetics. For many of these drugs, there is literature evidence that supports their potential metabolism by microbial β-glucuronidases. For example, many opioids (including morphine [62,63], codeine [62,63], hydromorphone [64,65], nalmefene [66], ketobemidone [67], and buprenorphine [68]) are reported to be glucuronidated in the liver and reactivated by β-glucuronidases. Estrogens are also known to form conjugates with glucuronides and could be reactivated by β-glucuronidase as well [69–73]. NSAIDs such as naproxen is metabolized by glucuronidation and is reactivated in the gut by microbial β-glucuronidases [74]. This reactivation causes intestinal damage, which can be alleviated using β-glucuronidase inhibitors [74]. This is also the case with other NSAIDs such as diclofenac, ketoprofen, and indomethacin [59,75,76]. Lorazepam and oxazepam are among the benzodiazepines that were found to be glucuronidated and reactivated by β-glucuronidase [77].

In addition to all previously mentioned drugs, several others listed in S1 Data were reported to be affected by β-glucuronidase, including irinotecan [11,78,79], regorafenib [61], tamoxifen [80], mitiglinide [81], clozapine [82], and zidovudine [83]. In summary, among the 100 drugs that we predicted to be metabolized by β-glucuronidases, we compiled evidence supporting the prediction for over 20 of them.

### Effects of the variation in the β-glucuronidase level

In this study, we analyzed the gut metagenome data from the HMP and found that the β-glucuronidase level was higher in the male gut metagenome than in the female counterpart. Interestingly, a few studies reported that opioids and NSAIDs are less effective in females than males [84,85]. Our results suggest that it may be (in part) because the β-glucuronidase level is lower in female gut metagenomes than in their male counterparts, which may lower the bioavailability of the drugs in females. Furthermore, it was shown that indomethacin is more toxic in males than in females, this is could be due to the same reason [86]. If verified, our results can shed light on how the higher level of β-glucuronidase in males contributes to this observation as it can increase the bioavailability (and possible toxicity) of such medications more in males than in females. It would be necessary to consider this information in adjusting drug dosage in therapy due to the potential biological impact of the gut microbiome on the reactivation of medications-glucuronide conjugates [71,84,85].

We also showed that infant gut metagenomes (newborns and one-year-old infants but not four-month-old infants) have a higher β-glucuronidase level than those of their mothers (Fig 7). We speculate that a few factors are involved in driving this observation—breastfeeding cessation and bilirubin. First, in Bäckhed study [26], most mothers ceased breastfeeding their infants at one year of age. Cessation of breastfeeding—but not solid food intake—drove the composition and function of the gut microbiome of infants [26]. However, the introduction of new food items that are glucuronidated is another possible driver for the increase in the level of microbial β-glucuronidases in one-year-old infants in comparison with that of four-month-olds, because microbial β-glucuronidases can use these glucuronidated molecules as substrates. This is further supported by the fact that β-glucuronidase is present in breast milk [49,50]. In the absence of breast milk, the gut microbiota may compensate for the decrease with breastfeeding cessation by increasing its expression of β-glucuronidases. Second, we speculate that bilirubin is one of the main drivers for the high levels of β-glucuronidase in newborns’ gut metagenomes, in comparison with that of 4-month-old infants. This is because bilirubin levels escalate during the first few days after birth [87–89] and bilirubin is metabolized through glucuronidation as well [90]. Thus, the increase in the production of bilirubin and its glucuronide conjugate possibly induces microbial β-glucuronidases. This hypothesis is supported by previous findings [91,92], in which a spike in microbial β-glucuronidases resulted after the intake of substrates to the enzymes.

Because β-glucuronidase is involved in the breakdown of bilirubin, it was suspected to play a role in the development of hyperbilirubinemia in infants [46–50]. The activity of β-glucuronidase in the infant gut is important because this enzyme can hydrolyze bilirubin-glucuronide conjugate, facilitating the reabsorption of bilirubin and causing hyperbilirubinemia unless it is further metabolized by the gut microbiome [46–50]. Further experimental studies are needed to investigate β-glucuronidase and bilirubin-degrading enzymes in the infant gut to determine the susceptibility of infants to develop hyperbilirubinemia. This apparent variability is based on SRA data and needs to be experimentally confirmed to show variability in the β-glucuronidase activity as well.

### Factors affecting the gut β-glucuronidase activity

It is noteworthy that β-glucuronidases from different bacterial species vary in their expression and/or activity, which are critical factors to consider besides the enzyme level assessed in our study [93]. For instance, *Clostridium perfringens* produces 34-fold higher β-glucuronidase activity than *Escherichia coli* when grown in human bile [93]. In addition, β-glucuronidases are a diverse group of enzymes that exhibit various degrees of specificity to their substrates due to differences in their protein structure, folding, and/or active sites [12,94,95]. For example, *Bacteroides uniformis* produces three different β-glucuronidases that metabolize SN-38-glucuronide, a metabolite of irinotecan, at different rates [95]. Moreover, inhibitors used to reduce β-glucuronidases activity vary in their inhibitory capacity toward each of those three enzymes [95]. Therefore, our approach provides a preliminary indication of the capacity of the gut microbiome to produce β-glucuronidases and needs to be complemented with other mentioned analyses. If the observed variability in the β-glucuronidase level in the human gut metagenomes is proven to be biologically significant, this will lead to the variation among individuals in the metabolism of the 100 medications in S1 Data. This will contribute to our understanding of the drug response variability and lead to improved drug dose optimization.

Evidence supporting our predictions includes a significant portion of the literature documenting that over 20 of those 100 medications (S1 Data) are metabolized through glucuronidation, then reactivated by β-glucuronidases. A concrete case is already evident for the chemotherapeutic agent, irinotecan, due to the severity of the interaction [10,11,78]. Another strong evidence was recently presented regarding some of the NSAIDs (e.g., diclofenac, ketoprofen, and indomethacin), in which their metabolites were found to be reactivated by gut microbial β-glucuronidases, causing intestinal toxicity [59,75,76]. Such toxicity was alleviated by using β-glucuronidase inhibitors. In the case of irinotecan and NSAIDs, the effect of β-glucuronidase on drug metabolism is severe. However, other drug interactions could be less severe, thus undocumented. Other medications with evidence for their metabolism by β-glucuronidases include important drugs in the market, ranging from general pain medications to specialized medications such as chemotherapeutic agents. Our approach gives a head start to research in the field of pharmacomicrobiomics, as it provides a hypothesis for each drug in S1 Data, regarding their metabolism and possible metagenome interaction depending on the level of β-glucuronidase. These hypotheses need to be tested experimentally to prove or disprove them.

### *Bacteroides* as a possible major contributor to the β-glucuronidase level

*Bacteroides* genus belongs to the Bacteroidetes phylum. Several of its species are human pathogens, while many others are part of the human commensal microbiota [96]. *Bacteroides* species harbors many glycosyl hydrolases including several β-glucuronidases that alter nutrients and xenobiotics availability in the gut [97]. Our analysis revealed that only *Bacteroides*—among 200 genera—is positively correlated with the β-glucuronidase level in the HMP gut metagenomes (Fig 8). Therefore, we suggest that *Bacteroides* can be a key player in the production of gut microbial β-glucuronidases and their influence upon the metabolism of glucuronidated drugs. This is supported by other research groups, who showed a similar correlation. For instance, Molan et al. showed that the ingestion of blackcurrant products reduces the *Bacteroides* population in humans, as well as the activity of gut β-glucuronidase [98]. Yip et al. showed that administration of tacrine, a reversible cholinesterase inhibitor, in rats increases the abundance of *Bacteroides* and the β-glucuronidase gene count [92]. This was associated with enhanced enterohepatic recycling of the deglucuronidated drug leading to hepatotoxicity [92]. Finally, Son et al. used the interleukin 10 (IL-10) knockout mice, an animal model of inflammatory bowel disease, and showed that *Bacteroides* was decreased in IL-10 knockout female mice [99]. This was associated with a decrease in the β-glucuronidase gene [99]. At the molecular level, advances to uncover the regulation of β-glucuronidase expression were beautifully conducted by Little et al. [100]. One of their findings was that different drug-glucuronide conjugates differentially bind to β-glucuronidase regulator, thus influencing β-glucuronidase expression [100].

### Choice of reference β-glucuronidase sequences

Appropriate reference β-glucuronidase sequences are required to accurately identify hit sequences from metagenomic data. In a recent analysis of the gut metagenomes from 139 individuals, Pollet et al. [12] explained a potential issue arising from selecting reference sequences based on protein domain information from the Pfam database. Because both β-glucuronidase and β-galactosidase have glycosyl hydrolase domains, the Pfam database groups these two proteins into a single protein family of glycosyl hydrolases, making it difficult to distinguish β-glucuronidase from β-galactosidase. To correctly identify β-glucuronidase, Pollet et al. [12] checked if a protein sequence had the "N-K" motif (consisting of invariant Gly, Asn, and Lys residues), which is unique to the active site of β-glucuronidase. These considerations led to the selection of four reference sequences (from *Escherichia coli*, *Clostridium perfringens*, *Streptococcus agalactiae*, and *Bacteroides fragilis*) in the study by Pollet et al.

In our study, the sixty sequences of the protein family PRK10150 from CDD [35,36] were used as reference sequences. CDD contains a collection of well-annotated multiple sequence alignment NCBI-curated domains as well as those imported from major sources of protein domains, including Pfam, Simple Modular Architecture Research Tool (SMART), Clusters of Orthologous Groups of proteins (COG), Protein Clusters (PRK), and The Institute for Genomic Research's database of protein families (TIGRFAMs) [35,36]. The protein family PRK10150 used in our study did not suffer from the issue mentioned in Pollet et al [12]. All PRK10150 sequences were specifically annotated as β-glucuronidases and had the N-K motive, distinct for β-glucuronidase.

With that said, it may be interesting to investigate the similarity of the sixty PRK10150 sequences with the four reference sequences used in Pollet et al. [12] Therefore, when the four sequences from Pollet et al. were used as a query to search CDD using the CD-Search tool [35,36]., PRK10150 was returned as the most specific hit, indicating that they do belong to this protein family. This suggests that the PRK10150 sequences are appropriate as reference sequences in our analysis.

### Limitations of the present study

While our study focuses on the quantification of the overall β-glucuronidase level in the gut, there are two additional things to consider to accurately estimate the effects of the gut β-glucuronidase level upon the drug metabolism.

First, β-glucuronidases from different microbial species have varying activities for a given substrate. For example, Pollet al. [12] demonstrated that β-glucuronidase from *E*. *coli* breaks down p-nitrophenol glucuronide (PNPG) very efficiently, with a rate ten-fold greater than that from *B*. *fragilis*. However, when tested in the same study with a large polysaccharide substrate (haparin nonasaccharide), β-glucuronidase from β-glucuronidase exhibited no activity, contrary to those from other microbes including *B*. *fragilis* [12].

Second, β-glucuronidase from a given microbial species has a distinct substrate preference. For example, Dashnyam et al. [15] demonstrated that while β-glucuronidase from *E*. *coli* can process a wide range of substrates, β-glucuronidase from *B*. *fragilis* can process large substrates more efficiently. The study of Dashnyam et al. [15] also tested the β-glucuronidase activity against the glucuronide conjugates of four drugs identified in our study (namely, diclofenac, SN-38 [irinotecan toxic metabolite], acetaminophen, and morphine). Interestingly, *B*. *fragilis* β-glucuronidase was more active against the drug-conjugates in the following order: diclofenac > SN-38 > acetaminophen, with no apparent activity against morphine [15].

These two factors are not taken into account in our approach presented here because of the lack of relevant data. In essence, it requires cataloging information on species-specific β-glucuronidases and their drug specificity, which is beyond the scope of this study. We believe that an opportunity for improvement will arise soon as more data in this area are made available through public repositories. It is also noteworthy that our study focused on quantifying the overall level of microbial β-glucuronidases in the “unassembled” metagenome, while Pollet et al. gave more emphasis on evaluating the differences in the structures and functions of the microbial proteins in the “assembled” metagenome. While a direct comparison between our study and the study by Pollet et al. is not straightforward because of several important differences (e.g., the data sets employed, the reference sequences, the programs used, etc.), one important similarity exists: the assertion that the healthy human gut microbiota harbors a great variability in their encoded β-glucuronidase sequences.

## Conclusions

Our novel analysis of human metagenomes is a robust approach to explore the metabolic capacity of the human microbiome. This was achieved by harnessing the power of public information resources. The DrugBank database was used to find medications whose metabolism can be influenced by microbial β-glucuronidase. The variation of the microbiome-encoded β-glucuronidase level in the human gut was estimated using DIAMOND. We showed that the level of β-glucuronidase in the gut metagenomes from the HMP was higher in males than in females, which may influence the fate of 100 drugs, including morphine, estrogen, NSAIDs, benzodiazepines, and their structural analogues. We also showed that infant gut metagenomes at birth and 12 months of age have higher levels of β-glucuronidase than the metagenomes of their mothers. This is likely linked to the bilirubin level in infants and breastfeeding cessation, respectively. *Bacteroides* abundance was correlated with the level of β-glucuronidase. This research presents a new approach to predict metagenome-medication interactions as well as new hypotheses that need experimental testing. If confirmed, this can enable us to avoid potential drug-metagenome interactions. Our results complement the mounting evidence of the importance of the human microbiome in drug efficacy and safety. As additional metagenomic data becomes available, we may be able to predict potential metagenome-medication interactions and present therapeutic options that consider the role of human microbiomes in medication metabolism.

## Supporting information

S1 Data 100 medications that can be potentially affected by β-glucuronidases, along with their therapeutic categories, route of administrations, PubChem Compound ID (CID), and DrugBank ID. (XLSX)

S2 Data Structural similarity matrix of the 100 medications. (TXT)

S3 Data A list of the 1793 publicly available SRA data sets of gut metagenomes in the NCBI database that was used in this study. These comprise 997 males and 796 females. (TXT)

S4 Data A list of the 400 publicly available SRA data sets of gut metagenomes in the NCBI database that was used in this study. These comprise 300 samples for 100 infants (taken three times at birth, four months, and one year of age) and 100 samples for their mothers. (TXT)

S5 Data Non-default values for the parameters used in the NCBI SRA Toolkit and PRINSEQ++. (XLSX)

S6 Data Representative β-glucuronidase protein sequences used for alignment. (TXT)

S7 Data Spearman correlations between the β-glucuronidase level and the relative abundance of bacterial genera. In addition, example codes used in this study are available at GitHub [22].
(XLSX)
